# Durvalumab in Advanced Biliary Tract Cancer: Real‐World Data From a Large Cohort of Patients Across Multiple International Centers

**DOI:** 10.1111/liv.70428

**Published:** 2025-11-12

**Authors:** Margherita Rimini, Lorenzo Fornaro, Masafumi Ikeda, Oluseyi Abidoye, Jessica Lucchetti, Lorenzo Antonuzzo, Jin Won Kim, Federico Nichetti, Anna Saborowski, Tiziana Pressiani, Caterina Vivaldi, Ilario Giovanni Rapposelli, Frederik Peeters, Chiara Braconi, David J. Pinato, Emiliano Tamburini, Chiara Pircher, Stefano Tamberi, Florian Castet, Monica Verrico, Alessandro Parisi, Nuno Couto, Hong Jae Chon, Andrea Martirena, Angela Lamarca, Matteo Landriscina, Fabian Fimkelmeier, Ester Oneda, Antonio Avallone, Emanuela Dell'Aquila, Lukas Perkhofer, Il Hwan Kim, Cidalia Maria de Sousa Pinto, Ingrid Garajova, Beodeul Kang, Salvatore Corallo, Elisabetta Fenocchio, Giovanni Farinea, Alessandro Pastorino, Anna Diana, Yoo Changhoon, Marta Schirripa, Maria Grazia Rodriquenz, Mario Scartozzi, Lucrezia Zumstein, Michele Ghidini, Gerald W. Prager, Giuseppe Aprile, Gian Paolo Spinelli, Su Yung‐Yeh, Stephen Chan, Emily Warmington, Alessia Lancianese, Tanios Bekaii‐Saab, Daniele Lavacchi, Minsu Kang, Sara Lonardi, Arndt Vogel, Alessandra Anna Prete, Silvia Bozzarelli, Francesca Salani, Mara Persano, Gianluca Masi, Rita Balsano, Monica Niger, Silvia Camera, Laura Passeri, Michele Ferrara, Tomoyuki Satake, Lorenza Rimassa, Andrea Casadei‐Gardini, Lo Prinzi Federica, Silvana Leo, Nicola Personeni, Grazia Rodriguez, Ana Rolo, Cecilia Alvim Moreira, Roque Jricardo

**Affiliations:** ^1^ Vita‐Salute San Raffaele University Milan Italy; ^2^ IRCCS San Raffaele Hospital Milan Italy; ^3^ Unit of Medical Oncology 2, Azienda Ospedaliero‐Universitaria Pisana Pisa Italy; ^4^ Department of Hepatobiliary and Pancreatic Oncology National Cancer Center Hospital East Kashiwa Japan; ^5^ Department of Internal Medicine Mayo Clinic Phoenix Arizona USA; ^6^ Fondazione Policlinico Universitario Campus Bio‐Medico Roma Italy; ^7^ Clinical Oncology Unit, Careggi University Hospital Florence Italy; ^8^ Department of Experimental and Clinical Medicine University of Florence Florence Italy; ^9^ Division of Hematology/Medical Oncology, Department of Internal Medicine, Seoul National University Bundang Hospital Seoul National University College of Medicine Seongnam‐si Republic of Korea; ^10^ Oncology 1 Unit, Department of Medical Oncology Veneto Institute of Oncology IOV—IRCCS Padua Italy; ^11^ Department of Gastroenterology, Hepatology, Infectious Disease and Endocrinology Hannover Medical School Hannover Germany; ^12^ Medical Oncology and Hematology Unit Humanitas Cancer Center, IRCCS Humanitas Research Hospital Milan Italy; ^13^ Department of Translational Research and New Technologies in Medicine and Surgery University of Pisa Pisa Italy; ^14^ Department of Medical Oncology IRCCS Istituto Romagnolo per lo Studio dei Tumori (IRST) “Dino Amadori” Meldola Italy; ^15^ Digestive Oncology University Hospitals Leuven Leuven Belgium; ^16^ Beatson West of Scotland Centre, CRUK Scotland Centre, University of Glasgow (School of Cancer Sciences) Glasgow UK; ^17^ Department of Surgery & Cancer, Imperial College London Hammersmith Hospital London UK; ^18^ Division of Oncology, Department of Translational Medicine University of Piemonte Orientale Novara Italy; ^19^ Department of Oncology and Palliative Care Cardinale G. Panico, Tricase City Hospital Tricase Italy; ^20^ Department of Medical Oncology Fondazione IRCCS Istituto Nazionale dei Tumori Milan Italy; ^21^ Santa Maria Delle Croci Hospital, AUSL Romagna Ravenna Italy; ^22^ Department DIMEC University of Bologna Bologna Italy; ^23^ Upper Gastrointestinal and Endocrine Tumor Unit Vall d'Hebron Institute of Oncology (VHIO), Vall d'Hebron University Hospital Barcelona Spain; ^24^ UOC Oncologia A, Department of Hematology, Oncology and Dermatology Policlinico Umberto I, Sapienza University of Rome Rome Italy; ^25^ Clinica Oncologica e Centro regionale di Genetica Oncologica Università Politecnica delle Marche, Azienda Ospedaliero‐Universitaria delle Marche Ancona Italy; ^26^ Digestive Unit, Champalimaud Clinical Centre, Champalimaud Research Centre Lisbon Portugal; ^27^ Division of Medical Oncology, Department of Internal Medicine CHA Bundang Medical Center, CHA University School of Medicine Seongnam Korea; ^28^ Department of Medical Oncology OncoHealth Institute, Fundacion Jimenez Diaz University Hospital Madrid Spain; ^29^ HealthResearch Institute, Fundacion Jimenez Diaz University Hospital (IIS‐FJD) Madrid Spain; ^30^ Universidad Autonoma de Madrid (UAM) Madrid Spain; ^31^ Medical Oncology and Biomedical Therapy, Policlinico Riuniti Foggia Italy; ^32^ Medical Clinic 1, University Hospital, Goethe‐University Frankfurt Frankfurt am Main Germany; ^33^ Dipartimento di Oncologia Medica Fondazione Poliambulanza Brescia Italy; ^34^ Clinical Experimental Abdominal Oncology Unit Istituto Nazionale Tumori‐IRCCS Fondazione G. Pascale Naples Italy; ^35^ IRCCS Istituto Nazionale Tumori Regina Elena Roma Italy; ^36^ Internal Medicine 1, University Hospital Ulm Ulm Germany; ^37^ Institute of Molecular Oncology and Stem Cell Biology, Ulm University Hospital Ulm Germany; ^38^ Division of Oncology, Department of Internal Medicine Haeundae Paik Hospital, Inje University College of Medicine Busan Republic of Korea; ^39^ Department of Medical Oncology Unidade Local de Saude do Algarve (ULSAlg) Faro Portugal; ^40^ Medical Oncology Unit, University Hospital of Parma Parma Italy; ^41^ Medical Oncology Unit, IRCCS Policlinico San Matteo, Department of Internal Medicine and Medical Therapy University of Pavia Pavia Italy; ^42^ Division of Medical Oncology Candiolo Cancer Institute IRCCS Turin Italy; ^43^ Department of Oncology University of Turin, San Luigi Hospital Turin Italy; ^44^ Medical Oncology IRCCS Ospedale Policlinico San Martino Genova Italy; ^45^ Oncology Unit Ospedale del Mare Napoli Italy; ^46^ ASAN Medical Center, University of Ulsan College of Medicine Seoul Korea; ^47^ Medical Oncology Unit, Department of Oncology and Hematology Belcolle Hospital Viterbo Italy; ^48^ Oncology Unit Fondazione IRCCS Casa Sollievo della Sofferenza San Giovanni Rotondo Italy; ^49^ Medical Oncology University and University Hospital of Cagliari Cagliari Italy; ^50^ GI Oncology Unit and Familial Cancer Unit, Oncology Department Hospital Universitario 12 de Octubre Madrid Spain; ^51^ Fondazione IRCCS Ca'Granda Ospedale Maggiore Policlinico Milan Italy; ^52^ Department of Medicine I, Clinical Division of Oncology Medical University Vienna Vienna Austria; ^53^ Department of Medical Oncology AULSS 8 Berica Vicenza Italy; ^54^ UOC Oncologia Territoriale, Polo Pontino La Sapienza Università Di Roma Latina Italy; ^55^ National Institute of Cancer Research National Health Research Institutes Tainan Taiwan; ^56^ Department of Oncology National Cheng Kung University Hospital, College of Medicine, National Cheng Kung University Tainan Taiwan; ^57^ Department of Clinical Oncology Prince of Wales Hospital, The Chinese University of Hong Kong Hong Kong China; ^58^ Long Family Chair in Liver Cancer Research, Division of Gastroenterology and Hepatology Toronto General Hospital, Medical Oncology, Princess Margaret Cancer Centre, Schwartz Reisman Liver Research Centre Toronto Canada; ^59^ Department of Biomedical Sciences Humanitas University Milan Italy

**Keywords:** biliary tract cancer, chemotherapy, cholangiocarcinoma, cisplatin, durvalumab, gemcitabine, immunotherapy

## Abstract

**Background:**

We recently published the first real‐world multicenter and multi‐institutional study of cisplatin, gemcitabine, and durvalumab in patients with advanced biliary tract cancer (BTC). Here we present an expanded patient cohort with an increased sample size and longer median follow‐up.

**Methods:**

The study population included patients with advanced BTC, who received cisplatin/gemcitabine plus durvalumab at 55 centers across 12 countries in Europe, the United States, and Asia. The primary endpoints of the study were progression‐free survival (PFS) and overall survival (OS). Secondary endpoints were overall response rate (ORR) and safety.

**Results:**

Overall, 1358 patients were enrolled. Median PFS was 7.6 months (95% CI: 7.2–8.1), and median OS was 15.6 months (95% CI: 14.8–16.5). ORR was 35.6%, and DCR was 82.7%. Any grade AEs occurred in 1213 patients (89.3%). Grade 3–4 AEs occurred in 597 patients (43.2%). The rate of immune‐related AEs (irAE) was 20.3%. Grade 3–4 irAE occurred in 3.0% of patients. At the multivariate analysis for OS, normal albumin level (HR 0.68, 95% CI 0.57–0.81, *p* < 0.0001), CEA levels within normal ranges (HR 0.68, 95% CI 0.57–0.82, *p* < 0.0001), NLR < 3 (HR 0.62, 95% CI 0.52–0.74, *p* < 0.0001), ECOG PS 0 (HR 0.51, 95% CI 0.42–0.61, *p* < 0.0001), and prior surgery (HR 0.80, 95% CI 0.65–0.99, *p* = 0.036) were positive prognostic factors.

**Conclusion:**

The updated findings, derived from an expanded cohort, further support the adoption of durvalumab in combination with gemcitabine and cisplatin in routine clinical practice, reinforcing the efficacy and safety outcomes demonstrated in the phase III TOPAZ‐1 trial.

**Summary**

- This study included over 1300 patients with advanced biliary tract cancer treated with chemotherapy and the immunotherapy durvalumab worldwide.
- The treatment proved effective and safe, with survival similar to that seen in clinical trials.
- These findings support its use as a standard first‐line option for advanced biliary tract cancer.

## 1 Introduction

Biliary tract cancers (BTC) represent a heterogeneous group of malignancies characterised by aggressive biological behaviour, poor prognosis, and limited therapeutic options [1, 2, 3, 4, 5, 6, 7]. In recent years, significant advances have been made, with several new therapeutic strategies introduced into the treatment landscape for advanced disease. Two randomised phase 3 trials led to the introduction of immunotherapy in the therapeutic armamentarium for advanced BTC [8, 9, 10, 11]. In particular, both the anti‐programmed cell death ligand 1 (anti‐PD‐L1) agent durvalumab and the anti‐programmed cell death 1 (anti‐PD‐1) agent pembrolizumab were approved as first‐line therapy in combination with the chemotherapy backbone of cisplatin and gemcitabine, according to the TOPAZ‐1 and KEYNOTE‐966 trials [8, 9, 10, 11]. Concurrently, several targeted therapies have gained approval for molecularly selected subgroups of previously treated patients, including Ivosidenib for those with *IDH1* mutations [12, 13], pemigatinib and futibatinib for those with *FGFR2* fusions or rearrangements [14, 15], and zanidatamab for HER2 overexpressed tumours [16]. In this rapidly evolving therapeutic landscape, the evaluation of real‐world data (RWD) has become critically important. First, RWD allows confirmation of clinical trial findings in patients commonly treated in everyday clinical practice. Second, there remains an urgent need to identify those patients who are more likely to respond and gain a survival benefit with the combination of immunotherapy and chemotherapy. In this context, investigations based on real‐world cohorts constitute a valuable complement to data generated by randomised clinical trials. Recently, our group demonstrated the feasibility and effectiveness of the cisplatin, gemcitabine, and durvalumab combination in the real‐world setting, first in a national Italian cohort [17], and subsequently in a broader, international cohort [18]. The latter study included 666 patients treated across 39 centers in 11 countries and reported a median progression‐free survival (PFS) of 8.2 months and a median overall survival (OS) of 15.1 months. The overall response rate was 32.7%, with a disease control rate of 45.2%. These results closely mirror those of the TOPAZ‐1 trial and further support the clinical benefit and generalizability of chemoimmunotherapy with durvalumab in patients with advanced biliary tract cancer. In the present analysis, we expanded the cohort to over 1300 patients by involving additional institutions worldwide, updating our previous database to further validate the results of the TOPAZ‐1 trial in a large, multicenter, multinational real‐world population with advanced BTC.

## 2 MATHERIAL and Methods

### 2.1 Study Population

The study population included patients with unresectable, locally advanced, or metastatic BTC, including intrahepatic (iCCA), perihilar extrahepatic cholangiocarcinoma (pCCA), distal extrahepatic cholangiocarcinoma (dCCA), and gallbladder carcinoma (GBC). Data were retrospectively collected from 55 sites across 12 countries (Italy, Germany, Austria, Spain, Portugal, Belgium, United Kingdom, United States (US), Republic of Korea, China, Hong Kong Special Administrative Region of China, Japan). Patients included in the analysis received first‐line treatment with cisplatin (25 mg/m^2^ on days 1 and 8), gemcitabine (1000 mg/m^2^ on days 1 and 8), and durvalumab (1500 mg on day 1), all administered intravenously on a 21‐day cycle for up to eight cycles in the setting of standard of care practice. This was followed by durvalumab monotherapy every 4 weeks until disease progression or the occurrence of unacceptable toxicity.

The present study was approved by the local Ethics Committee at each center, complied with the provisions of the Good Clinical Practice guidelines and the Declaration of Helsinki and local laws, and fulfilled Regulation (EU) 2016/679 of the European Parliament and of the Council of 27 April 2016 on the protection of natural persons with regard to the processing of personal data.

### 2.2 Statistical Analysis

The objective of the present study was the confirmation of TOPAZ‐1 data in real‐world practice, in a population from both Eastern and Western countries. The primary endpoints were PFS and OS achieved with the combination of durvalumab plus cisplatin and gemcitabine in a real‐world cohort of patients. Secondary endpoints were overall response rate (ORR) and safety.

PFS was defined as the time from the date of treatment initiation to the date of disease progression or death or last follow‐up whichever occurred first. OS was defined as the time from the date of treatment initiation to the date of death. Survival curves were estimated using the product‐limit method of Kaplan–Meier. PFS and OS were reported as median values expressed in months, with a 95% confidence interval (CI).

ORR was assessed by the investigator and defined as the proportion of patients who achieved complete response (CR) or partial response (PR); disease control rate (DCR) was defined as the proportion of patients who achieved ORR or stable disease (SD). Treatment response was evaluated by computed tomography (CT) and categorised as CR, PR, SD, or progressive disease (PD) by local review according to Response Evaluation Criteria in Solid Tumours (RECIST) 1.1.

Adverse events (AEs) were graded according to the National Cancer Institute Common Terminology Criteria for Adverse Events, version 5.0.

Unadjusted and adjusted hazard ratios (HRs) by baseline characteristics were calculated using the Cox proportional hazards model. Categorical variables were compared using the Fisher exact test. Survival medians and rates were evaluated using the Kaplan–Meier method. A *p* value < 0.05 was considered statistically significant.

The hazard functions for OS and PFS were estimated using kernel density estimation (KDE) with a bandwidth parameter set to 0.2 to account for the variability in event timing. This approach allows for a non‐parametric estimation of the instantaneous risk of an event over time, providing a more flexible representation of the hazard rate compared to traditional parametric models. The hazard functions were calculated based on the observed event times, and the resulting curves were plotted to visualise the temporal dynamics of risk.

A MedCalc package (MedCalc version 20.2) was used for statistical analysis.

## 3 Results in the Cisplatin, Gemcitabine, Durvalumab Cohort

### 3.1 Outcome

From February 2022 to April 2025, 1358 patients were identified at 55 sites across 12 countries.

Patient demographics and disease characteristics are reported in Table 1.

**TABLE 1:** Baseline demographic and clinical characteristics of patients with advanced biliary tract cancer treated with first‐line cisplatin, gemcitabine, and durvalumab across 55 international centers.

Characteristic	*N* (%)
Male	711 (52.4%)
Female	647 (47.6%)
Gallbladder	234 (17.2%)
iCCA	758 (55.8%)
dCCA	149 (11.0%)
pCCA	217 (16.0%)
No surgery	972 (71.6%)
Yes surgery	386 (28.4%)
No stent or biliary dreinage	908 (70.7%)
Stent	264 (20.5%)
External dreinage	82 (6.4%)
Both	31 (2.4%)
ECOG‐PS 0	682 (50.4%)
ECOG‐PS > 0	672 (49.6%)
ca19.9 NV	441 (34.0%)
ca19.9 no NV	856 (66.0%)
CEA NV	694 (56.8%)
CEA no NV	528 (43.2%)
NLR < 3	565 (46.5%)
NLR ≥ 3	651 (53.5%)
Albumin NV	806 (73.3%)
Albumin no NV	237 (22.7%)
Bilirubin NV	1038 (79.3%)
Bilirubin no NV	271 (20.7%)
AST NV	530 (51.3%)
AST no NV	505 (48.7%)
ALT NV	800 (70.6%)
ALT no NV	271 (29.4%)
	Median (Min‐Max)
Age	69 (24–92)
Monocytes	0,61 (0–6.5)
Platelets	250 (24–753)
ALP	154 (21–3629)
GGT	135 (12–3647)

Abbreviations: ALT, alanine aminotransferase; AST, aspartate aminotransferase; BTC, biliary tract cancer; CA 19‐9, carbohydrate antigen 19‐9; CEA, carcinoembryonic antigen; dCCA, distal cholangiocarcinoma; ECOG PS, Eastern Cooperative Oncology Group performance status; GBC, gallbladder cancer; iCCA, intrahepatic cholangiocarcinoma; NLR, neutrophil‐to‐lymphocyte ratio; pCCA, perihilar cholangiocarcinoma.

At data cutoff (May 1, 2025), the median duration of follow‐up was 14.5 months (95% CI: 13.7–36.5), 934 patients (68.8%) discontinued treatment due to disease progression, and 605 patients (44.5%) had died.

**FIGURE 1:**
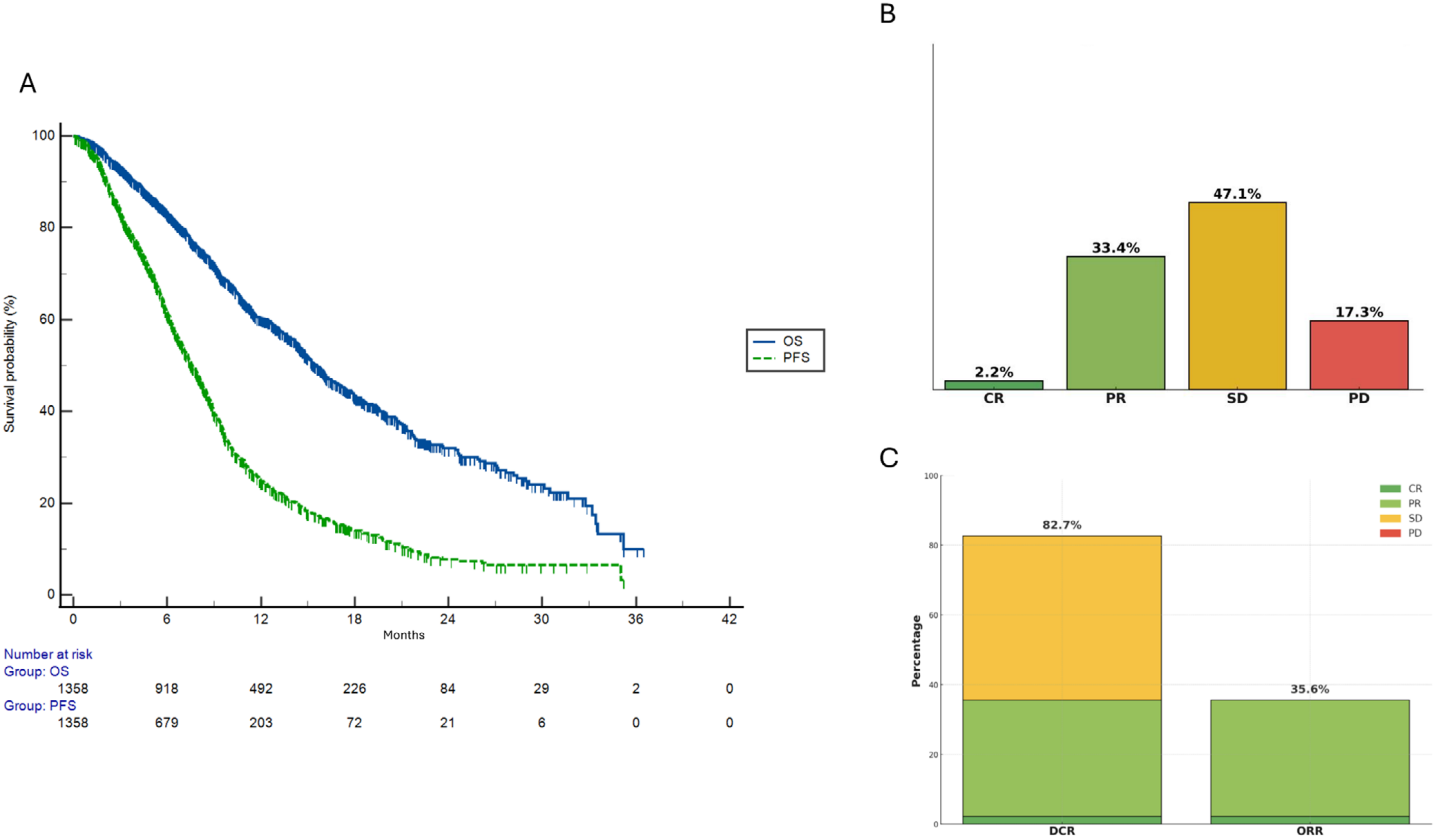
(A) Kaplan–Meier curves for overall survival and progression‐free survival in the cohort of patients with advanced biliary tract cancer treated with cisplatin, gemcitabine, and durvalumab. (B) Distribution of best overall responses to treatment: Complete response (CR), partial response (PR), stable disease, and progressive disease. (C) Overall response rate (ORR) and disease control rate. BTC, biliary tract cancer; CR, complete response; DCR, disease control rate; ORR, overall response rate; OS, overall survival; PD, progressive disease; PFS, progression‐free survival; PR, partial response; SD, stable disease.

**FIGURE 2:**
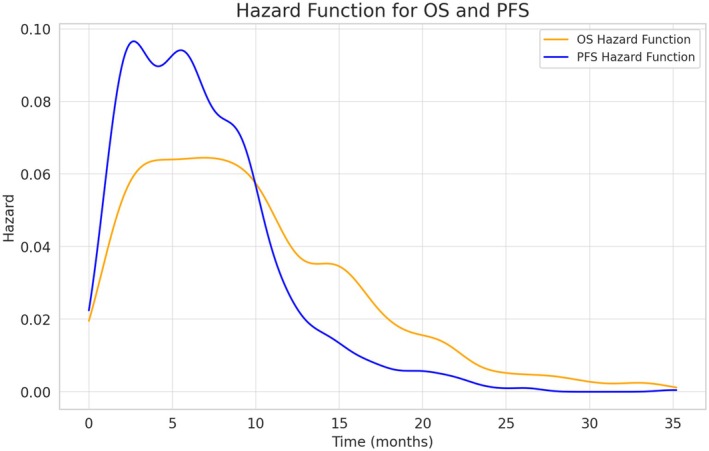
Hazard functions for OS and PFS in the cohort of patients treated with cisplatin, gemcitabine, and durvalumab. Hazard estimates were calculated using kernel density estimation. KDE, kernel density estimation; OS, overall survival; PFS, progression‐free survival.

Estimated median PFS was 7.6 months (95% CI: 7.2–8.1), and median OS 15.6 months (95% CI: 14.8–16.5) (Figure 1A). PFS rates at 6, 12, 18, and 24 months were 61.9%, 25.7%, 15.5%, and 10.0%, respectively, while the OS rates at the same time points were 83.4%, 60.5%, 43.6%, and 32.7%, respectively. The estimated hazard function for PFS demonstrated an initial rapid increase, peaking at approximately 3 months, followed by a progressive decline. The peak hazard for PFS reached a maximum of 0.098 per month, gradually decreasing to < 0.01 per month beyond 24 months. In contrast, the hazard function for OS showed a broader peak, reaching a maximum of 0.073 per month between 6 and 12 months, with a subsequent decline to < 0.01 per month after 30 months (Figure 2).

ORR was 35.6%. The percentage of patients achieving a CR was 2.2%, while PR was 33.4% and SD was 47.1%, leading to a DCR of 82.7% (Figure 1B,C).

**FIGURE 3:**
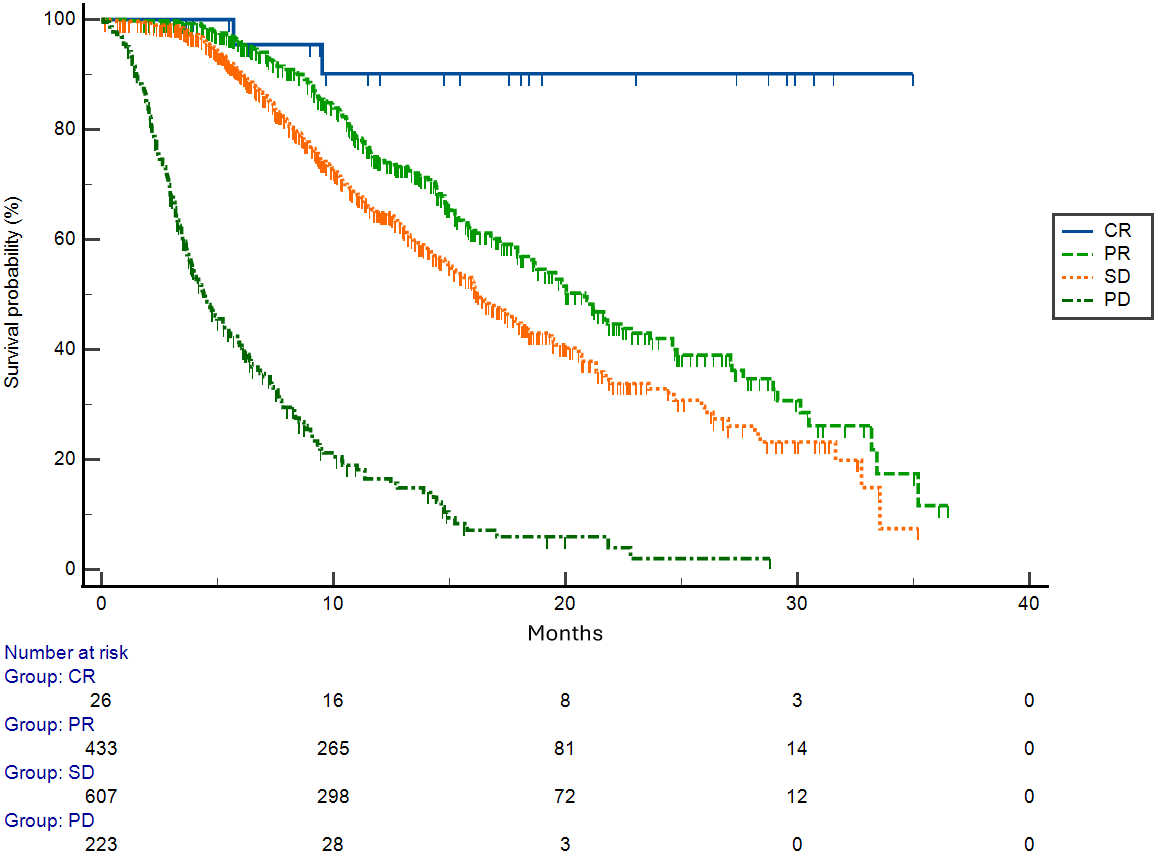
Overall survival according to best response to treatment: CR, PR, SD, and PD. Kaplan–Meier curves are shown for each response subgroup. CR, complete response; OS, overall survival; PD, progressive disease; PR, partial response; SD, stable disease.

**FIGURE 4:**
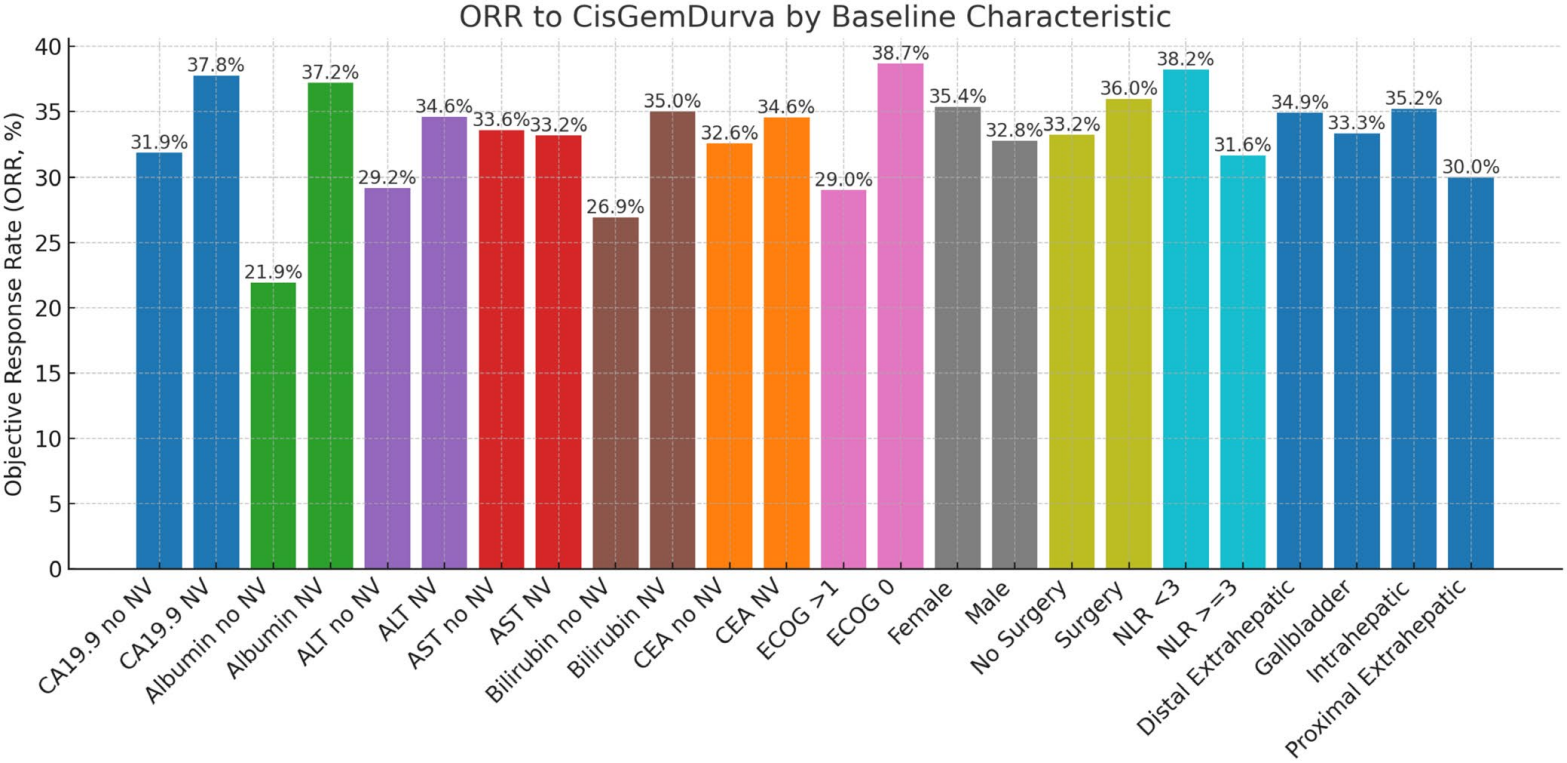
ORR to cisplatin/gemcitabine plus durvalumab by baseline characteristics. ALT, alanine aminotransferase; AST, aspartate aminotransferase; CA 19‐9, carbohydrate antigen 19‐9; CEA, carcinoembryonic antigen; ECOG PS, Eastern Cooperative Oncology Group performance status; NLR, neutrophil‐to‐lymphocyte ratio; ORR, objective response rate.

Median OS was not reached for patients achieving CR, while it was 20.8 months (95% CI, 18.6–22.8) for those with PR, 16.3 months (95% CI, 15.1–18.0) for patients with SD, and 4.4 months (95% CI, 3.7–5.5) for those with PD (Figure 3). The estimated OS rates at 6, 12, 18, and 24 months were 92.5%, 83.7%, 83.7%, and 83.7%, respectively, for patients with CR; 96.0%, 75.0%, 57.2%, and 42.4% for those with PR; 90.3%, 65.1%, 45.0%, and 34.0% for patients with SD; and 40.7%, 16.3%, 6.8%, and 4.1% for those with PD, respectively. The Figure 4 shows the ORR by the baseline characteristics in the whole population.

**FIGURE 5:**
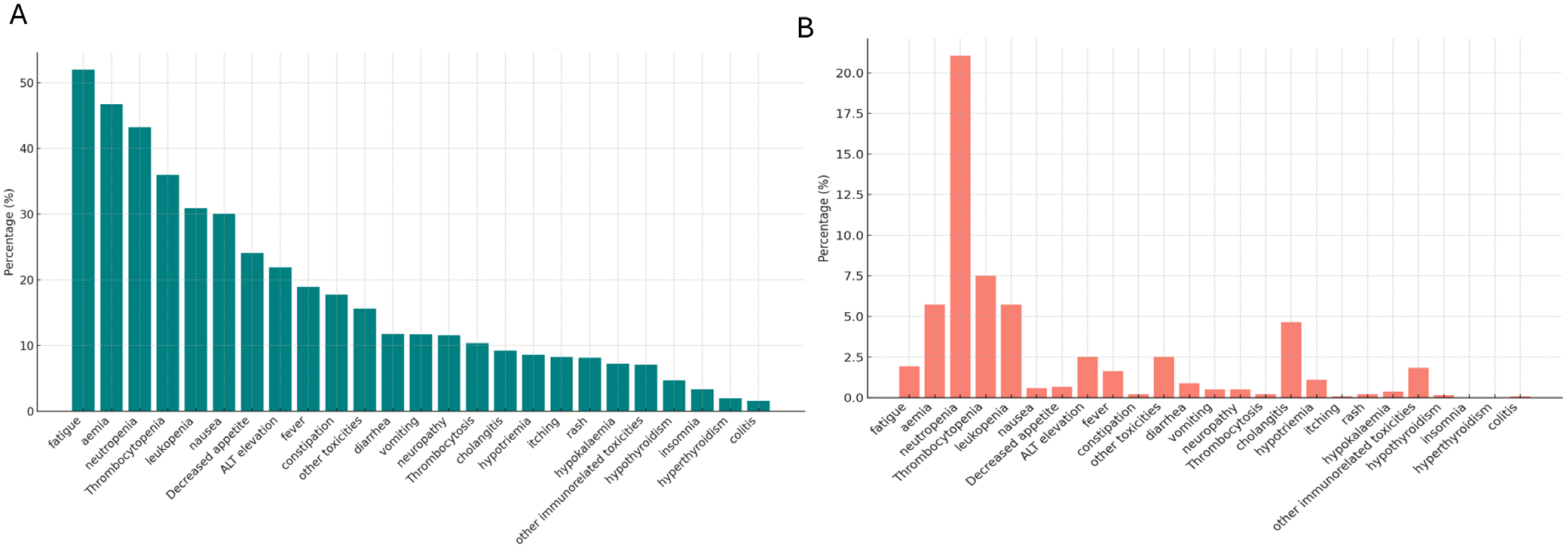
(A) Frequency of any grade adverse events (AEs) in patients treated with cisplatin, gemcitabine, and durvalumab. (B) Frequency of grade 3–4 AEs. AE, adverse event.

Any grade AEs occurred in 1213 patients (89.3%) (Figure 5A). Grade 3–4 AEs occurred in 597 patients (43.2%) (Figure 5B). The most common AEs were fatigue (52.0%), anaemia (46.7%), neutropenia (43.2%), and thrombocytopenia (35.9%). Grade 5 treatment‐related AEs (TRAEs) occurred in 4 patients (0.4%; 2 cholangitis and 2 febrile neutropenia), none of them related to immunotherapy. The rate of immune‐related AEs (irAE) was 20.3%. Grade 3–4 irAE occurred in 3.0% of patients. The most common AEs were rash (8.1% all grade; 0.2% grade > 2), itching (8.2% all grade; 0.1% grade > 2), hypothyroidism (4.7% all grade; 0.1% grade > 2), hyperthyroidism (2.0% all grade; 0% grade > 2), and colitis (1.5% all grade; 0.1% grade > 2). The rate of discontinuation of durvalumab due to AEs was 1.7%. Figure 5 provides a summary of the observed toxicities.

### 3.2 Subgroup Analysis

In the univariate analysis, ECOG PS 0 (HR 0.60, 95% CI 0.52–0.68, *p* < 0.0001), albumin levels > 3.5 g/dL (HR 0.63, 95% CI 0.52–0.77, *p* < 0.0001), CA 19–9 levels within normal ranges (HR 0.75, 95% CI 0.66–0.86, *p* = 0.0001), CEA levels within normal ranges (HR 0.65, 95% CI 0.56–0.75, *p* < 0.0001), NLR < 3 (HR 0.67, 95% CI 0.58–0.76, *p* < 0.0001), ALT levels within normal ranges (HR 0.75, 95% CI 0.63–0.89, *p* = 0.0014), AST levels within normal ranges (HR 0.77, 95% CI 0.67–0.90, *p* = 0.0009), and bilirubin levels within normal ranges (HR 0.83, 95% CI 0.70–0.98, *p* = 0.03), pCCA vs. iCCA (HR 0.72, 95% CI 0.61–0.86) and prior surgery (HR 0.78, 95% CI 0.68–0.90, *p* = 0.0006) were associated with improved PFS.

In the multivariate analysis for PFS, albumin levels within normal ranges (HR 0.82, 95% CI 0.71–0.94, *p* = 0.0068), CA 19‐9 levels within normal ranges (HR 0.80, 95% CI 0.68–0.93, *p* = 0.0056), CEA levels within normal ranges (HR 0.83, 95% CI 0.72–0.96, *p* = 0.0138), NLR < 3 (HR 0.73, 95% CI 0.64–0.85, *p* < 0.0001), ECOG PS 0 (HR 0.61, 95% CI 0.51–0.72, *p* < 0.0001), pCCA vs. iCCA (HR 0.86, 95% CI 0.80–0.93, *p* = 0.0002), and prior surgery (HR 0.79, 95% CI 0.68–0.93, *p* = 0.0056) were highlighted as protective prognostic factors.

In the univariate analysis for OS, albumin levels within normal ranges (HR 0.39, 95% CI 0.30–0.50, *p* < 0.0001), CA 19‐9 levels within the normal ranges (HR 0.70, 95% CI 0.59–0.83, *p* < 0.0001), CEA levels within normal ranges (HR 0.51, 95% CI 0.43–0.61, *p* < 0.0001), NLR < 3 (HR 0.54, 95% CI 0.46–0.64, *p* < 0.0001), bilirubin levels within the normal ranges (HR 0.59, 95% CI 0.48–0.73, *p* < 0.0001), AST levels within normal ranges (HR 0.73, 95% CI 0.60–0.87, *p* = 0.0009), ALT levels within normal ranges (HR 0.74, 95% CI 0.59–0.91, *p* = 0.0062), ECOG PS 0 (HR 0.44, 95% CI 0.38–0.52, *p* < 0.0001), pCCA vs. iCCA (HR 0.74, 95% CI 0.59–0.92), and prior surgery (HR 0.79, 95% CI 0.68–0.93, *p* = 0.0056) were associated with improved OS.

In the multivariate analysis for OS, albumin levels within normal ranges (HR 0.68, 95% CI 0.57–0.81, *p* < 0.0001), CEA levels within normal ranges (HR 0.68, 95% CI 0.57–0.82, *p* < 0.0001), NLR < 3 (HR 0.62, 95% CI 0.52–0.74, *p* < 0.0001), ECOG PS 0 (HR 0.51, 95% CI 0.42–0.61, *p* < 0.0001), and prior surgery (HR 0.80, 95% CI 0.65–0.99, *p* = 0.036) were highlighted as prognostic factors.

**FIGURE 6:**
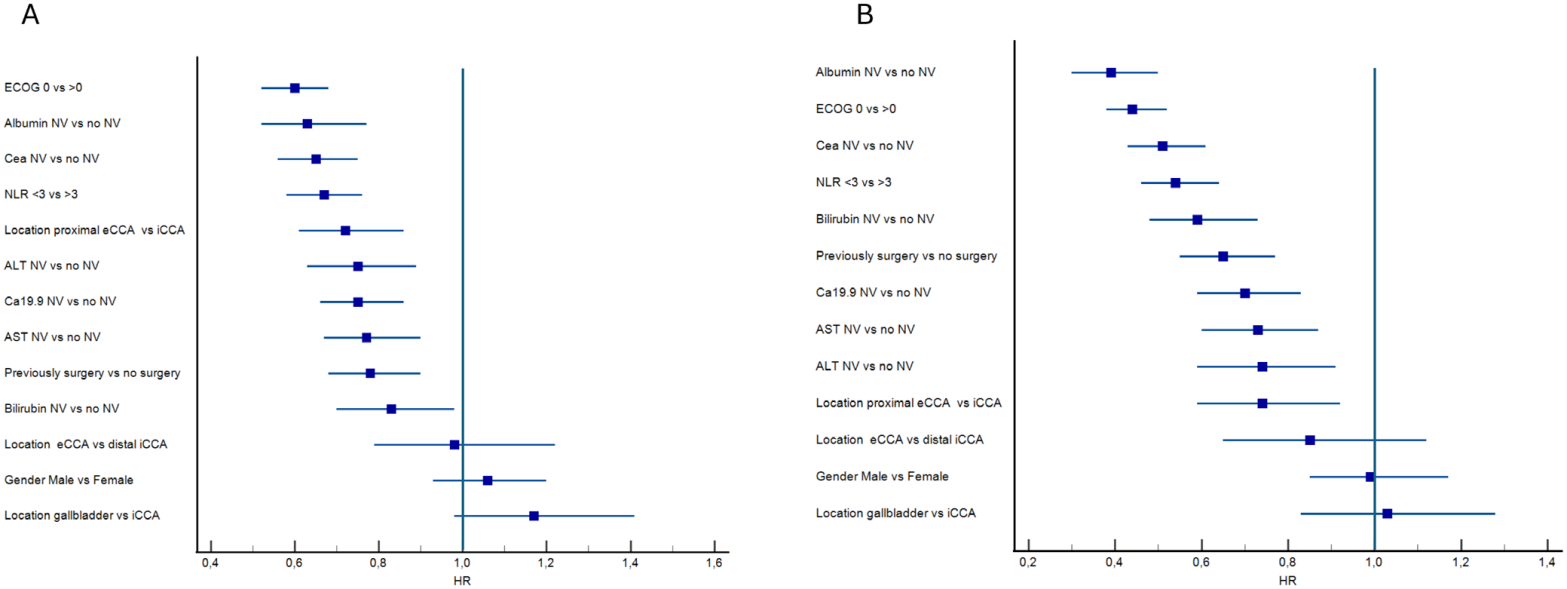
Forest plots from subgroup analyses showing hazard ratios (HRs) for (A) progression‐free survival (PFS) and (B) overall survival (OS). HR, hazard ratio; OS, overall survival; PFS, progression‐free survival.

Figure 6 summarises the forest plots for PFS in Figure 5A and OS in Figure 5B.

## 4 Discussion

To the best of our knowledge, this study reports the largest global real‐world cohort of patients with advanced BTC treated with cisplatin, gemcitabine, and durvalumab. It represents an updated analysis of the cohort previously published in 2024 [16], expanded in terms of both patient numbers and duration of follow‐up. Data were collected from 55 institutions across Europe, the US, and Asia. After a median follow‐up of 14.5 months, the median OS was 15.6 months (95% CI 14.8–16.5), with OS rates at 6, 12, 18, and 24 months of 83.4%, 60.5%, 43.6%, and 32.7%, respectively, confirming the survival benefit of chemoimmunotherapy in this setting. The updated results of the TOPAZ‐1 trial, after a median follow‐up of 41.3 months, showed a median OS with the chemo‐immunotherapy combination of 12.9 months (95% CI 11.6–14.1); OS rates at 12, 18, 24 and 36 months were 54.3% (95% CI 48.8–59.4), 34.8% (95% CI 29.6–40.0), 23.6% (95% CI 18.7–28.9) and 17.0% (95% CI 11.0–18.6), respectively [10]. Moreover, looking at survival outcomes according to best response, in the present analysis median OS was not reached, 20.8 months (95% CI 18.6–22.8), 16.3 months (95% CI 15.1–18.0) and 4.4 months (95% CI 3.7–5.5) in patients achieving CR, PR, SD, and PD, respectively. Despite the inherent limitations of comparing prospective randomised phase III trials with retrospective real‐world analyses, the survival outcomes observed in the present study, conducted in an unselected population, are comparable to those achieved in the TOPAZ‐1 trial. These findings suggest that even in a broader, less selected real‐world cohort, patients with BTC can derive similar benefit to the one reported in clinical trials from the addition of durvalumab to cisplatin and gemcitabine. This further supports the use of this chemoimmunotherapy combination as a valid first‐line treatment option in clinical practice. Interestingly, the estimated hazard function for PFS demonstrated a rapid initial increase, peaking at around 3 months, followed by a gradual decline to less than 0.01 per month beyond 24 months. For OS, the hazard peaked between 6 and 12 months and then progressively decreased, reaching below 0.01 per month after 30 months. The shape of the hazard function for both PFS and OS highlights a critical early‐risk phase, particularly within the first 3–12 months. Patients who remain progression‐free or alive beyond this interval appear to enter a phase of sustained disease control or prolonged survival, suggesting the presence of a clinically meaningful long‐term responder subgroup. The prolonged OS benefit for a subgroup of patients and the emergence of a survival plateau observed with immunotherapy are supported by a biological rationale: unlike chemotherapy, which induces a transient cytotoxic effect, immune checkpoint inhibitors reactivate tumour‐specific T cells and promote the development of immunological memory, enabling long‐term tumour control even after treatment discontinuation [19, 20, 21, 22]. This durable immune activation underlies the delayed but sustained clinical benefit seen across multiple tumour types. A notable example is the HIMALAYA trial in advanced hepatocellular carcinoma, where the combination of durvalumab and the anti‐CTLA‐4 agent tremelimumab obtained a 5‐year OS rate of 19.6% [23], further confirming the capacity of immunotherapy to induce long‐lasting survival benefits, consistent with findings in Non–Small‐Cell Lung Cancer and melanoma [19, 20, 21].

In BTC, recent data from the TOPAZ‐1 trial suggest a similar trend, reflecting a separation of survival curves at around 6 months from treatment start and with a subset of patients obtaining prolonged benefit from the addition of durvalumab to chemotherapy [9].

In terms of responses, the present analysis is consistent with the phase III trial, with an ORR of 35.6% and a DCR of 82.7%.

In the current safety analysis we observed an overall incidence of AEs of 89.3%, slightly lower than in our previous study [17]. The spectrum of toxicities was generally consistent with prior findings [17], though slightly different from what was observed in the TOPAZ‐1 trial. In the present cohort, the most common AEs were fatigue, anaemia, neutropenia, and thrombocytopenia, whereas the TOPAZ‐1 trial primarily reported anaemia, nausea, constipation, and neutropenia. In both analyses, most relevant toxicities were related to the chemotherapy backbone. The slightly different toxicity profile observed in our analysis may partly be explained by the potential difficulties encountered in routine clinical practice when managing hematologic toxicities, which could be more challenging than handling subjective symptoms such as nausea or constipation. The incidence of grade 3–4 irAEs was consistent with that reported in the TOPAZ‐1 study, as was the proportion of patients who discontinued treatment due to adverse events (1.7%). These findings further support the manageable safety profile of this combination. The current analysis confirmed our previous findings in terms of prognostic factors. Of interest, in the multivariate analysis for PFS pCCA were associated with significantly longer PFS compared to iCCA in our cohort of patients (HR 0.86, 95% CI 0.80–0.93; *p* = 0.0002), consistent with data from the phase 3 trial. Indeed, a subgroup analysis of the TOPAZ‐1 trial demonstrated a larger PFS benefit from durvalumab added to gemcitabine/cisplatin in eCCA compared with iCCA (HR ~0.52 vs. 0.79) [8, 9]. These consistent trends suggest subtype‐specific differences in treatment response and disease biology that merit further investigation. In terms of prognostic impact in OS, albumin levels within normal ranges, prior surgery, low CEA levels, NLR < 3, and ECOG PS 0 were associated with longer OS. Albumin levels within normal ranges may reflect both the patients' nutritional status, and preserved liver function, both associated with prognosis in patients with advanced solid tumours [24].

Several reports have shown that prior surgery on the primary tumour is associated with improved survival outcomes [25, 26, 27, 28]. This may suggest that BTC recurring after radical resection could potentially display a less aggressive biological behaviour compared to tumours diagnosed de novo at an advanced or metastatic stage, although this remains a speculative hypothesis that warrants further investigation. Alternatively, it is possible that surgical intervention itself influences tumour biology by reducing overall tumour burden. Further investigations are needed to clarify underlying mechanisms associated with better prognosis observed in patients who underwent prior surgery.

Another noteworthy finding from the present analysis is that CEA emerged as a prognostic marker, whereas CA 19‐9 [29], traditionally more commonly used in the BTC setting, did not show a significant association with outcomes. This observation should be interpreted with caution, as CA 19‐9 remains the most widely validated biomarker in BTC and its prognostic role has been consistently confirmed in several large prospective and retrospective studies [29, 30].

Previously studies highlighted the role of CEA and CA 125 in the second‐line setting. Indeed, a post hoc analysis of the ABC‐06 trial showed that CA19‐9 levels measured at week 4 after chemotherapy initiation had limited prognostic value, whereas CEA and CA 125 demonstrated independent prognostic significance [31].

In addition, other previous retrospective analyses have shown the prognostic role of CEA in both surgical and advanced settings, particularly in iCCA. However, these studies consistently concluded that CEA levels are insufficient to define prognosis. Rather, CEA may provide greater clinical value when considered in combination with other prognostic factors [32, 33].

Our results therefore do not challenge the established relevance of CA 19‐9, but rather suggest that CEA may provide additional and complementary prognostic information, particularly when interpreted alongside CA 19‐9 and other clinical factors.

In addition, the present analysis confirmed the prognostic role of NLR, thus supporting its potential utility as a surrogate marker of the immune microenvironment [30]. In a recently published retrospective analysis of patients with BTC treated with chemo‐immunotherapy, Jin and colleagues developed a prognostic nomogram incorporating the number of treatment cycles, NLR, CEA levels, and the presence of bone metastases [34]. The findings of that study regarding the prognostic value of NLR and CEA are consistent with those of our analysis, but support the idea that integrating multiple clinical and laboratory parameters, such as CEA, CA 19‐9, and NLR, into composite prognostic models may offer a more effective approach to risk stratification than relying on individual markers alone. Further investigations are warranted to explore this approach.

The present study has several limitations. First, its retrospective design inherently carries the risk of selection bias. However, the large number of patients from multiple institutions across Eastern and Western countries strengthens the validity of the analysis. Moreover, a multivariate analysis was performed to mitigate potential confounding factors. The multicenter and multinational nature of the study enhances the generalizability of our findings but also introduces variability in clinical practice. In particular, the timing of tumour assessments during treatment was determined by individual physicians based on local protocols and practice patterns, which may have influenced the accuracy of the PFS data. The absence of a centralised radiologic review may have led to variability in response assessment across participating centers, although all sites adhered to RECIST 1.1 criteria and international standards for imaging evaluation.

Furthermore, the cohort was predominantly composed of patients from European centers, which may limit the generalizability of the findings to underrepresented geographic regions. Nevertheless, the inclusion of patients from Asia and the United States partially mitigates this imbalance and supports the global relevance of the observed outcomes.

Finally, the relatively short median follow‐up limits long‐term outcome evaluation; further follow‐up is warranted to confirm the observed results.

In conclusion, we present the largest global real‐world cohort of patients with advanced BTC treated with cisplatin, gemcitabine, and durvalumab, providing an updated analysis of the cohort previously published in 2024, now expanded in terms of the number of patients and follow‐up duration. The achieved results support the adoption of this regimen in clinical practice by reinforcing the efficacy and safety outcomes demonstrated in the phase III TOPAZ‐1 trial.

## Author Contributions

Conception and design: A. Casadei‐Gardini, M. Rimini, L. Rimassa, L. Fornaro. Acquisition of data (acquired and managed patients): All authors. Analysis and interpretation of data: A. Casadei‐Gardini, M. Rimini, L. Rimassa, L. Fornaro. Writing, review, and/or revision of the manuscript: A. Casadei‐Gardini, M. Rimini, L. Rimassa, L. Fornaro. Final approval of manuscript: All authors.

## Ethics Statement

The study was conducted in accordance with the Declaration of Helsinki and the protocol was approved by the Ethics Committee of each institution involved in the project. Under the condition of retrospective archival tissue collection and patients' data anonymization, our study was exempt from the acquisition of informed consent from patients by the institutional review board.

Institutional Review Board Statement: The Ethical Review Board of each Institutional Hospital approved the present study. This study was performed in line with the principles of the Declaration of Helsinki.

## Consent

Written informed consent for treatment was obtained for all patients.

## Conflicts of Interest

H.J.C.: received honoraria from Eisai, Roche, ONO, MSD, Bristol Myers Squibb, BeiGene, Sanofi, Servier, AstraZeneca, Aptamer Science, and Boryung; served on advisory boards for Eisai, Roche, ONO, MSD, Bristol Myers Squibb, BeiGene, Servier, AstraZeneca, Boryung, IMBDx, and Aptamer Science; and received research grants from Roche, BeiGene, Servier, IMBDx, Dong‐A ST, and Boryung Pharmaceuticals. F.C.: Roche, Servier; Honoraria: AstraZeneca, Eisai, Oncosil, Roche, Rovi, Servier. Travel and accommodation from Roche, Servier. N.C.: received speaker fees from AstraZeneca, Novartis, Servier. M.S.: advisory board and speakers' bureau: Amgen, Merck, Daiichi Sankyo, Astra Zeneca, Servier, BMS, Beigene. D.J.P.: Lecture fees: Bayer Healthcare, Astra Zeneca, EISAI, Bristol Myers Squibb, Roche, Boston. Scientific, Ipsen; Travel expenses: Bristol Myers‐Squibb, Roche, Bayer Healthcare, Astra Zeneca; Consulting fees: Mina Therapeutics, Galapagos, Parabilis, Boehringer Ingelheim, Ewopharma, EISAI, Ipsen, Roche, H3B, Astra Zeneca, DaVolterra, Mursla, Terumo, Avammune. Therapeutics, Starpharma, LiFT Biosciences, Exact Sciences; Research funding (to institution): MSD, BMS, GSK. I.G.R.: advisory board for AstraZeneca. L.R. received consulting fees from AbbVie, AstraZeneca, Basilea, Bayer, BMS, Eisai, Elevar Therapeutics, Exelixis, Genenta, Hengrui, Incyte, Ipsen, Jazz Pharmaceuticals, MSD, Nerviano Medical Sciences, Roche, Servier, Taiho Oncology, Zymeworks; lecture fees from AstraZeneca, Bayer, BMS, Eisai, Guerbet, Incyte, Ipsen, Roche, Servier; travel expenses from AstraZeneca and Servier; research grants (to Institution) from AbbVie, AstraZeneca, BeiGene, Exelixis, Fibrogen, Incyte, Ipsen, Jazz Pharmaceuticals, MSD, Nerviano Medical Sciences, Roche, Servier, Taiho Oncology, TransThera Sciences, Zymeworks. A.C.‐G. reports consulting fees from AstraZeneca, Bayer, BMS, Eisai, Incyte, Ipsen, IQVIA, MSD, Roche, Servier; lecture fees from AstraZeneca, Bayer, BMS, Eisai, Incyte, Ipsen, Roche, Servier; travel expenses from AstraZeneca; research grants (to Institution) from AstraZeneca, Eisai. S.L.C. serves as an advisory member for AstraZeneca, MSD, Eisai, BMS, Ipsen, and Hengrui, received research funds from MSD, Eisai, Ipsen, SIRTEX, and Zailab, and honoraria from AstraZeneca, Eisai, Roche, Ipsen, and MSD. T.P. received consulting fees from Bayer, Ipsen, and AstraZeneca; institutional research funding from Roche, Bayer, and AstraZeneca; travel expenses from Roche. C.B. received honoraria as a speaker (Astrazeneca, Incyte, Servier) and consultant (Incyte, Servier, Boehringer Ingelheim, Astrazeneca, Tahio, Jazz), received research funds (Avacta, Medannex, Servier) and her spouse is an employee of Astrazeneca. M.I. reports honoraria from AstraZeneca, Chugai Pharma, Eisai, Incyte, Lilly Japan, MSD, Novartis, Ono Pharmaceutical, Takeda, Teijin Pharma, Nihon Servier, Taiho and research funding from AstraZeneca, Bayer, Bristol‐Myers Squibb, Chiome Bioscience, Chugai, Eisai, Eli Lilly Japan, Delta‐Fly Pharma, Invitae, J‐Pharma, Merck biopharma, Merus N.V., MSD, Novartis, Nihon Servier, Ono, Syneos Health, and Rakuten Medical. G.W.P.: Advisories and/or Speaker fees: Servier, Bayer, Roche Amgen, Merck, MSD, BMS, Sanofi, Lilly, Astra Zeneca, Astellas, Pierre‐Fabre, Incyte, Arcus, CECOG. F.F. has received travel support from Ipsen, Abbvie, Astrazeneca and speaker's fees from AbbVie, MSD, Ipsen, Astrazeneca. L.P.: Advisory role: AstraZeneca, Servier, Travel expenses: AstraZeneca, Ipsen. S.L. reports research funding (to Institution) from Amgen, Astellas, Astra Zeneca, Bayer, Bristol‐Myers Squibb, Daiichi Sankyo, Hutchinson, Incyte, Merck Serono, Mirati, MSD, Pfizer, Roche, Servier; personal honoraria as invited speaker from Amgen, Astra Zeneca, Bristol‐Myers Squibb, Incyte, GSK, Lilly, Merck Serono, MSD, Pierre‐Fabre, Roche, Servier; participation in advisory board for Amgen, Astellas, Astra Zeneca, Bayer, Bristol‐Myers Squibb, Daiichi Sankyo, GSK, Incyte, Lilly, Merck Serono, MSD, Servier, Takeda, Rottapharm. J.D. received consulting fees and/or speaker honoraria from Amgen, AstraZeneca, Bayer, BMS, Eisai, Need Inc., Ipsen, Lilly, MediMix, Merck, MSD, Novartis, Roche and Servier. J.A. received consulting fees from AstraZeneca, Jazz Pharmaceuticals, MSD, Roche, Servier, Taiho Oncology, Zymeworks; lecture fees from AstraZeneca, Roche, Servier; travel expenses from AstraZeneca, Roche, Servier. A.D. Advisory Board: Amgen, Gentili, Invited Speaker: Eli Lilly, Novartis, Pfizer, Gentili, Amgen, Daiichi‐Sankyo, Roche, Gilead, Travel Support: Eli Lilly, Pfizer, Novartis, Ipsen, Gentili, Gilead, Editorial Collaboration: ACCMED. G.P.S. received advisory role/travel from Bayer, Roche, J&J, MSD, Novartis. M.D.R. received honoraria as a speaker from Astrazeneca. F.C. received speaker fees from AstraZeneca, Eisai, OncoSil, Roche, Rovi, Servier and travel and accommodation from Roche, Servier. M.N. received travel expenses from AstraZeneca, speaker honorarium from Accademia della Medicina, Incyte and Servier; honoraria from Sandoz, Medpoint SRL, Incyte, AstraZeneca and Servier for editorial collaboration. Consultant honoraria from EMD Serono, Basilea Pharmaceutica, Incyte, MSD Italia, Servier, Astrazeneca and Taiho. All remaining authors have declared no conflicts of interest.

## Data Availability

Data available on request from the authors.

## Appendix A

Members of the International BTC‐DURVA Investigators: Lo Prinzi Federica, Silvana Leo, Nicola Personeni, Grazia Rodriguez, Ana Rolo, Cecilia Alvim Moreira, Roque Jricardo.
